# Microemulsion Liquid Chromatographic Method for Simultaneous Determination of Simvastatin and Ezetimibe in Their Combined Dosage Forms

**DOI:** 10.1155/2013/132836

**Published:** 2013-10-27

**Authors:** Mohammed E. A. Hammouda, Mohamed A. Abu El-Enin, Dina T. El-Sherbiny, Dalia R. El-Wasseef, Saadia M. El-Ashry

**Affiliations:** Department of Medicinal Chemistry, Faculty of Pharmacy, University of Mansoura, Mansoura 35516, Egypt

## Abstract

A rapid HPLC procedure using a microemulsion as an eluent was developed and validated for analytical quality control of antihyperlipidemic mixture containing simvastatin (SIM) and ezetimibe (EZT) in their pharmaceutical preparations. The separation was performed on a column packed with cyano bonded stationary phase adopting UV detection at 238 nm using a flow rate of 1 mL/min. The optimized microemulsion mobile phase consisted of 0.2 M sodium dodecyl sulphate, 1% octanol, 10% n-propanol, and 0.3% triethylamine in 0.02 M phosphoric acid at pH 5.0. The developed method was validated in terms of specificity, linearity, lower limit of quantification (LOQ), lower limit of detection (LOD), precision, and accuracy. The proposed method is rapid (8.5 min), reproducible (RSD < 2.0%) and achieves satisfactory resolution between SIM and EZT (resolution factor = 2.57). The mean recoveries of the analytes in pharmaceutical preparations were in agreement with those obtained from a reference method, as revealed by statistical analysis of the obtained results using Student's *t*-test and the variance ratio *F*-test.

## 1. Introduction

**Figure 1:**
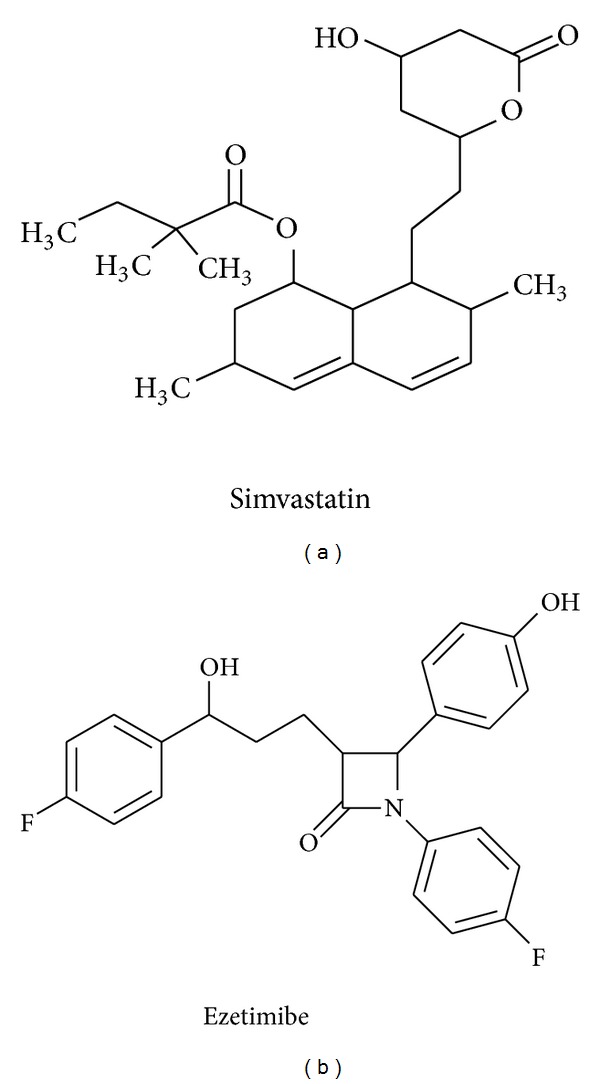
Structural formula of simvastatin and ezetimibe.

Simvastatin, 2,2-dimethylbutanoic acid (1S,3R,7S,8S,8aR)-1,2,3,7,8,8a-hexahydro-3,7-dimethyl-8-[2-[(2R,4R)-tetrahydro-4-hydroxy-6-oxo-2*H*-pyran-2-yl]ethyl]-1-naphthalenyl ester [1] (Figure 1(a)), is antihyperlipidemic drug, acting by competitive inhibition of 3-hydroxy-3-methylglutaryl coenzyme A reductase (HMG-CoA reductase), the rate determining enzyme for cholesterol synthesis in the liver [2]. Ezetimibe, (3R,4S)-1-(p-fluorophenyl)-3-[(3S)-3-(p-fluorophenyl)-3-hydroxypropyl]-4-(p-hydroxyphenyl)-2-azetidinone [2] (Figure 1(b)), is antihyperlipidemic agent, as it inhibits intestinal sterol absorption [2]. A combination dosage form containing simvastatin and ezetimibe was approved by FDA in July 2004 for the treatment hyperlipidemia.

Reviewing the literature revealed that several methods have been described for determination of SIM in pure forms as well as in pharmaceutical preparations, individually or in combination with other drugs. These methods include spectrophotometry [3–5], voltammetry [6], micellar enhanced kinetic chromatography (MEKC) [7], ultra performance liquid chromatography (UPLC) [8], and high performance liquid chromatography (HPLC) [9–12]. Regarding ezetimibe, various methods have been reported for its determination, individually or in combination with other drugs including spectrophotometry [13, 14], spectrofluorimetry [15], MEKC [16], gas chromatography-mass spectrometry (GC-MS) [17], UPLC [18], and HPLC [19–26]. Different methods were reported for the simultaneous determination of SIM and EZT in their coformulated tablets relying on spectrophotometric methods [27–30], MEKC [31], high performance thin layer chromatography (HPTLC) [32], and HPLC [33–35].

Microemulsions are clear, thermodynamically stable isotropic mixtures containing oil, water, surfactant, and most often also a medium chain alcohol acting as a cosurfactant. They can be considered as two-phase solvents consisting of a micellar phase surrounded by either an aqueous or an organic phase. The micellar phase may contain either an organic solvent or an aqueous phase (reversed micelles). Thus, the microemulsions may either be oil-in-water (o/w) or water-in-oil (w/o) microemulsions [36], where the o/w microemulsions are the preferred for HPLC. The partitioning and the interfacial adsorption of the analytes in the microheterogenous systems are responsible for the separations obtained [36]. In previous reports on microemulsion liquid chromatography (MELC) [37–48], the potential of application of microemulsions as mobile phases in LC analysis was proven. For SIM, MELC was reported for its quantitation either in the presence of impurities [40, 44] or its active metabolite [48].

### 1.1. The Objective of the Work

SIM and EZT are coformulated in medicinally recommended ratios of 2 : 1 and 4 : 1. Analysis of such mixture with strong spectral overlapping is challenging. Although there are a several methods for their simultaneous determination, it was the first time to use the microemulsion as a new mobile phase developed only in the last ten years and considered superior to aqueous mobile phase used by all reported methods [32–35], where it offers three simultaneous partitioning systems. The first system comprises microemulsion droplets/bulk of the eluent; the second comprises microemulsion droplets/stationary phase and; the third comprises, bulk of the eluent/stationary phase. Also it is the first time to use the cyano column as stationary phase which is adopted for the mixture separation instead of reversed stationary phase (C_18_ column) that has been used by other reported HPLC methods [33–35].

The proposed method present simple, rapid (retention time is 8.5 min), sensitive (LOD values were 0.15 and 0.17 *μ*g/mL for SIM and EZT, resp.), and efficient method for quantitation of the two drugs in their combined tablet dosage form compared with other methods which either; need more retention times “not less than 15–20 minutes” [33–35], and less sensitive [33–35], need complicated precaution like programmable detection [34], or finally need sophisticated instrumentation like MEKC [31]. Besides, the spectrophotometric methods [27–30] are less advanced and not considered a separation techniques.

## 2. Experimental

### 2.1. Materials and Reagents

 All the chemicals used were of analytical grade, and the solvents were of HPLC grade.

- Simvastatin and ezetimibe were kindly provided by Hikma Pharma S.A.E. (6th of October city, Cairo, Egypt), and both have purity of 99.9%. They were used as received without further purification.
- Alkor 20 plus tablets, batch no. 013 (Hikma Pharma S.A.E., 6th of October city, Cairo, Egypt), were purchased from commercial sources, labeled to contain 20 mg simvastatin and 10 mg ezetimibe.
- Alkor 40 plus tablets, batch no. 017 (Hikma Pharma S.A.E., 6th of October city, Cairo, Egypt), were purchased from commercial sources, labeled to contain 40 mg simvastatin and 10 mg ezetimibe.
- Sodium dodecyl sulphate (SDS) of 99% purity was obtained from Park Scientific Limited, Northampton, UK. 1-Propanol, methanol, and diisopropyl ether (HPLC grade) as well as triethylamine (TEA) were obtained from Riedel-de Häen (Seelze, Germany). 1-Butanol and tetrahydrofuran (HPLC grade) were obtained from Merck (Darmstadt, Germany). 1-Octanol (HPLC grade) was obtained from Aldrich (Gillingham, UK). 1-Butyl acetate was obtained from Fluka (Buchs, Switzerland). Orthophosphoric acid for analysis was obtained from Prolabo (Paris, France).

### 2.2. Apparatus

Separation was performed with Shimadzu LC-20A series chromatograph equipped with a 20 *μ*L Rheodyne injector valve and a SPD-20A UV detector operating at 238 nm (LC workstation, Nishinokyo-Kuwabara-cho, Nakagyo-Ku, Kyoto 604-8511, Japan). 

### 2.3. Columns and Mobile Phases

Separation was achieved on a Shim-pack cyano column (150 mm × 4.6 mm i.d., 5 *μ*m particle size 100 Å) from Shimadzu. The column was operated at ambient temperature. The components of the microemulsion were 0.2 M SDS, 10% 1-propanol, 1% 1-octanol, and 0.3% TEA in 0.02 M phosphoric acid; the pH was adjusted at 5. All the microemulsion components were mixed together and the pH was adjusted using TEA. Then, the mixture was treated on an ultrasonic bath for 30 min. The resulting transparent mobile phase was filtered through a 0.45 *μ*m membrane filter (Millipore, Ireland). Microemulsion was stable for at least 2 months [38].

### 2.4. Sample Preparation and Procedures

Stock solutions of SIM and EZT (100 *μ*g/mL) were prepared in methanol. The standard solutions were kept in the refrigerator and were found to be stable for at least 7 days.

#### 2.4.1. General Procedures and Calibration Graphs

To a set of 10 mL volumetric flasks, increasing volumes of the stock solutions of SIM and EZT were quantitatively transferred so as to give solutions within the concentration range of 0.5–40 and 1–50 *μ*g/mL, respectively, after being diluted to the volume with the microemulsion. Injection into the HPLC was performed at ambient temperature (25°C). Twenty-microliter aliquots were injected (in triplicate), and the calibration curves were constructed by plotting the area under the curve against the final concentration of both drugs. Alternatively, the corresponding regression equations were derived.

#### 2.4.2. Analysis of the Studied Drugs in Their Coformulated Tablets

Ten Alkor plus tablets were accurately weighed, finely pulverized, and thoroughly mixed. An accurately weighed amount of pulverized tablets equivalent to 20.0 mg SIM and 10.0 mg EZT (according to their pharmaceutical ratio in Alkor 20 plus) and 40.0 mg SIM and 10.0 mg EZT (according to their pharmaceutical ratio in Alkor 40 plus) was transferred into small conical flask and extracted with 3 × 30 mL of methanol. The extracts were collected then filtered into 100 mL volumetric flask. The conical flask was washed with few milliliters of methanol. The washings were passed into the same volumetric flask and completed to the volume with the same solvent. All samples were filtered through 0.45 *μ*m sample filters (RC25, Sartorius AG, Göttingen, Germany) prior to injection into the HPLC system. “The nominal content of the pharmaceutical preparation was calculated using the corresponding regression equation.”

## 3. Results and Discussion

**Figure 2:**
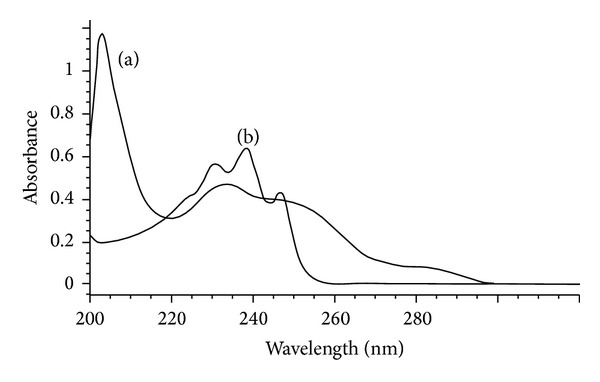
Absorption spectra of 20.0 *μ*g/mL EZT (a) and 20.0 *μ*g/mL SIM (b) in methanol.

The UV spectrum of SIM solution in methanol showed three absorption maxima at 231, 238, and 247 nm (Figure 2). Also EZT shows UV absorption maxima at 205, 233, and 250 nm (Figure 2). Thus, there is a great overlap in their absorption spectra, and hence, the conventional UV spectrophotometry cannot be used for their simultaneous determination. The proposed method permitted satisfactory resolution between the two drugs (resolution factor (*R*_*s*_) = 2.57 and selectivity factor (*α*) = 1.69) in a reasonable time of less than 8.5 min. The retention times for SIM and EZT were 5.5 and 8.2 min., respectively. The proposed method offers high sensitivity as about 0.15 *μ*g/mL of SIM and 0.17 *μ*g/mL of EZT could be detected accurately. It also permitted the quantification of the drugs in pure form as well as in coformulated tablets.

### 3.1. Method Development

**Figure 3:**
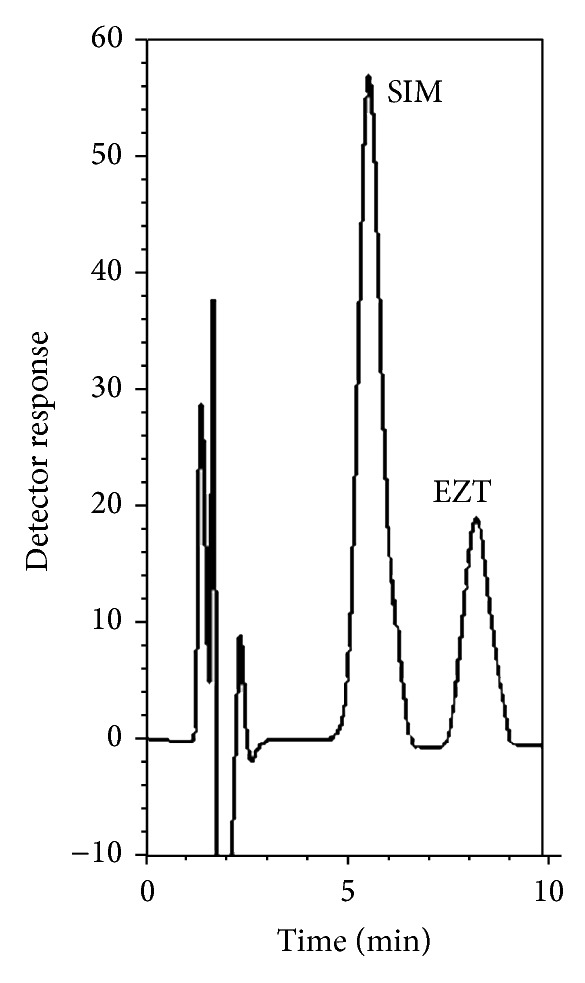
Typical chromatogram for the separation of SIM (40 *μ*g/mL, 5.5 min) and EZT (20 *μ*g/mL, 8.2 min) using microemulsion mobile phase. Chromatographic system: column, cyano (5 *μ*m) 150 mm × 4.6 mm. Mobile phase microemulsion, 0.2 M SDS, 10% n-propanol, 1% n-octanol, and 0.3% triethylamine, in 0.02 M phosphoric acid, pH 5. Flow rate, 1 mL/min; UV detection at 238 nm; column temperature, ambient.

An optimum separation of the two drug substances, with a resolution factor of 2.57, was achieved using a mobile phase consisting of 0.2 M SDS, 10% 1-propanol, 1% 1-octanol, and 0.3% TEA in 0.02 M phosphoric acid of pH 5.0 in a reasonable time of less than 8.5 min, with maximum detector response.  Figure 3 represents the obtained chromatogram of synthetic mixture containing 40 and 20 *μ*g/mL of SIM and EZT, respectively. The different parameters affecting the separation selectivity of the MELC system were investigated and optimized.

### 3.2. The Stationary Phase

Separation was attempted on two different columns: a LiChrosorb RP-18 column and a Shim-pack cyano column. The experimental studies revealed that the cyano column was the suitable one since symmetric peaks with reasonable resolution were obtained (*t*_*R*_ = 5.5 min and 8.2 min for SIM and EZT, resp.). Meanwhile, the use of C_18_ column was found to be suitable as it shows asymmetric peaks and bad resolution (*R*_*s*_ = 1.1).

### 3.3. The Mobile Phase

#### 3.3.1. The Concentration of the Surfactants

**Figure 4:**
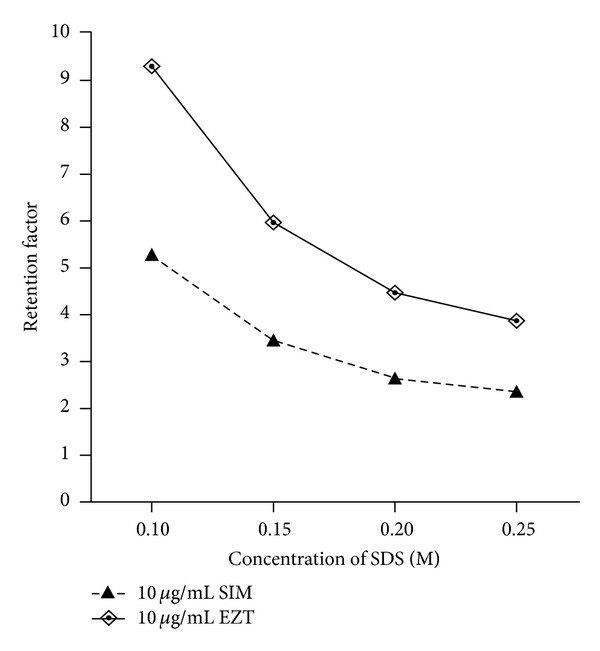
Effect of molar concentration of surfactant (SDS) on the retention factors of SIM 10 *μ*g/mL and EZT 10 *μ*g/mL using microemulsion mobile phases consisting of different concentrations of SDS, 10% n-propanol, 1% n-octanol, 0.3% triethylamine, in 0.02 M phosphoric acid, pH 5.

The effect of SDS concentration on retention time was investigated using microemulsions containing SDS concentrations ranging from 0.10 to 0.25 M. It was found that an increase in the concentration of SDS decreased the retention time of both drug substances over the investigated range due to their distribution into the increased volume of the microemulsion droplets or to the surface of the droplets which run with the speed of the mobile phase (Figure 4). A concentration of 0.20 M was found to be suitable for routine use as it provides good separation efficiency and the best retention factors for both drugs.

#### 3.3.2. The Effect of Cosurfactant

The effect of cosurfactant concentration was investigated over the concentration range of 5–15%. It was found that increasing the cosurfactant concentration results in decreasing the retention times of the two drugs; this could be attributed to increasing the proportion of organic phase in the microemulsion (Table 1). 5% propanol was unsuitable for separation due to overlap of the two peaks; 10% propanol was selected as optimum concentration as it provided good resolution and reasonable retention factors as indicated in Table 1. The cosurfactant nature greatly influences the mobile phase behavior, and changing the type of the cosurfactant can alter the selectivity [38–42]. 10% propanol was replaced with tetrahydrofuran, 1-butanol, or acetonitrile in an attempt to study the effect of the nature of the cosurfactant on the selectivity and efficiency of separation. Acetonitrile produces insufficient separation, while the use of butanol as cosurfactant results in bands broadening and peaks retardation especially in case of EZT. Tetrahydrofuran provided poor resolution and lower NETP when compared with propanol as cosurfactant which provided the highest resolution, good retention factor, and highest NETP for both drugs (Table 1).

#### 3.3.3. The Effect of pH

**Figure 5:**
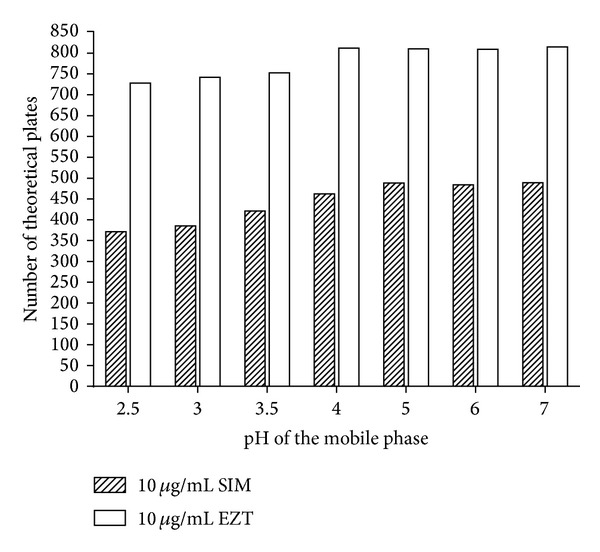
Effect of pH on the number of theoretical plates of SIM 10 *μ*g/mL and EZT 10 *μ*g/mL using microemulsion mobile phases consisting of 0.2 M SDS, 10% n-propanol, 1% n-octanol, and 0.3% triethylamine, in 0.02 M phosphoric acid, at different pH.

The pH of the mobile phase was changed in intervals from 2.5 to 7 using increasing amounts of triethylamine in phosphoric acid. It was found that the retention time of SIM and EZT was not significantly affected by changing the pH. The two drugs differ in their dissociation constants as expressed by their p*Ka* values, where SIM has p*Ka* value of 0 [49] and EZT has p*Ka* value of 9.66 [1]. On the other hand, both drugs have nearly equal hydrophobicity as expressed by their log *P* (octanol/water), where SIM has log *P* value of 4.68 [1] and EZT has log *P* value of 4.39 [1]. Thus, EZT is still unionized all over the selected pH range in contrast to SIM which will be fully ionized in this range, and hence, their separation depends on the difference in their p*Ka* values, where the more polar ionized drug SIM is eluted faster than the unionized less polar drug EZT. In this study, a pH value of 5 seemed to be optimal for the separation and detection of both analytes in a short run with satisfactory resolution (*R*_*s*_ = 2.57) and good separation efficiency as indicated by NETP (Figure 5). 

#### 3.3.4. The Internal Organic Phase

A micellar mobile phase identical to the microemulsion system but without the internal phase n-octanol was investigated. It was found that the resolution of the peaks as well as NETP were decreased. Three different organic solvents 1-octanol, butyl acetate, and diisopropyl ether were tested as internal organic phases (using 1% concentration) so as to present a range of polarity. The molecular volume of the oil, relative to the hydrophobic chain of the surfactant, affects the extent to which it penetrates the surfactant tails of the oil water interface. It was found that the separation could be successfully achieved using each of the three solvents. However, 1-octanol seemed to be optimal for separation and detection of both analytes because it provides the best resolution in a reasonable run time and the highest NETP (Table 1).

The effect of flow rate on separation of peaks of the studied compounds was studied in the range of 0.6–1.4 mL/min. A flow rate of 1 mL/min was optimal for good separation in a reasonable time (less than 8.5 min).

## 4. Validation of the Method

The developed MELC method was subjected to method validation according to ICH Q2(R1) guidelines [50]. The following parameters were considered: linearity, sensitivity, LOD, LOQ, specificity, accuracy, and precision.

### 4.1. Linearity

Linear relationships were established for both drugs by plotting the area under the curve against each drug concentration. The concentration ranges were found to be 0.5–40 *μ*g/mL and 1–50 *μ*g/mL for SIM and EZT, respectively. 

Linear regression analysis of the data by proposed method gave the following equations:

(1) $$\begin{array}{c} \text{PA}=-3.07+68.35C\;\left(r=0.9999\right)\;\;\text{for}\;\text{SIM},\\ \text{PA}=-10.55+41.93C\;\left(r=0.9999\right)\;\;\text{for}\;\text{EZT}, \end{array}$$

where PA is the peak area, *C* is the concentration of the drug (*μ*g/mL), and *r* is the correlation coefficient. Statistical analysis of the data gave high values of the correlation coefficient (*r*) of the regression equations, small values of the standard deviation of residuals (*S*_*y*/*x*_), intercepts (*S*_*a*_), and slopes (*S*_*b*_), and small values of the percentage of relative standard deviation and the percentage of relative error (Table 2). These data point to low scattering of points around the calibration curve and the high accuracy and precision of the proposed method.

### 4.2. Limit of Quantification (LOQ) and Limit of Detection (LOD)

LOQ and LOD were calculated according to ICH Q2(R1) recommendations [50] using the following equations:

(2) $$\begin{array}{c} \text{LOQ}=10\frac{S_{a}}{b},\;\;\text{LOD}=3.3\frac{S_{a}}{b}, \end{array}$$

where *S*_*a*_ is the standard deviation of the intercept and *b* is the slope of the calibration curve.

LOQ values were found to be 0.47 and 0.53 *μ*g/mL, while LOD values were found to be 0.15 and 0.17 *μ*g/mL for SIM and EZT, respectively.

### 4.3. Accuracy and Precision

To prove the accuracy of the proposed method, the results of the assay of SIM and EZT were compared with those of the reference method [30]. Statistical analysis of the results using Student's *t*-test and variance ratio *F*-test [51] revealed no significant difference between the performance of the two methods regarding the accuracy and precision, respectively (Table 3). The reference method depends on the simultaneous determination of SIM and EZT by ratio derivative spectrophotometric method, where the ratio first-derivative amplitudes were measured at 242.5 nm (^1^D_242.5_) and 299.5 nm (^1^D_299.5_) for the determination of SIM and EZT, respectively [30]. The intraday and interday precisions and accuracy of proposed MELC method were examined by triplicate analysis of SIM at three different concentrations of 10.0, 20.0, and 30.0 *μ*g/mL and EZT at 20.0, 30.0, and 40.0 *μ*g/mL in one day and for three consecutive days. The precision of the proposed method was satisfactory, as indicated by the low values of SD and RS; also the low values of % Er indicate good accuracy of the method (Table 4).

### 4.4. Specificity

The specificity of the method was investigated by observing any interference encountered from the presence of common tablet excipients that are present in coformulated tablets, including butylated hydroxyanisole, citric acid monohydrate, croscarmellose sodium, hypromellose, lactose monohydrate, magnesium stearate, microcrystalline cellulose, and propyl gallate. These excipients did not interfere with the proposed method.

### 4.5. Applications

#### 4.5.1. Analysis of SIM/EZT in Synthetic Mixtures and Coformulated Tablets

The proposed method was applied to the simultaneous determination of SIM and EZT in synthetic mixtures in the medicinally recommended ratios of 2 : 1 and 4 : 1. Furthermore, the proposed method was successfully applied for their determination in coformulated tablets in the same ratios. The results shown in Tables 5 and 6 are in good agreement with those obtained using the reference method [30]. Statistical analysis of the results obtained using Student's *t*-test and variance ratio *F*-test [51] revealed no significant difference between the performance of the two methods regarding the accuracy and precision, respectively.

## 5. Conclusion

A new reliable and specific HPLC method for the simultaneous determination of SIM and EZT in pure form and synthetic mixture as well as in pharmaceutical preparations using microemulsion as mobile phase with UV detection has been developed. The method has a short turnover time (8.5 min), and the LOD and RSD values are sufficiently good for the applicability of this method for quality control laboratories with HPLC availability. Moreover, microemulsion mobile phase provided an additional advantage over aqueous mobile phase, where it offers alternative partitioning mechanisms due to the presence of surfactant and oil droplets. 

## Figures and Tables

**Table 1:** Effect of different experimental parameters on the retention factors, number of theoretical plates, and resolution.

Parameter	Retention factors (*D*_*m*_)		NETP		*R*_*s*_
	SIM	EZT	SIM	EZT	
Concentration of cosurfactant %					
5.0	Overlapped peaks				
7.5	3.22	4.87	311	587	1.96
10.0	**2.64**	**4.47**	**488**	**810**	**2.57**
12.5	2.10	4.14	545	859	3.03
15.0	2.08	4.21	584	882	3.10
Type of cosurfactant					
Propanol	**2.64**	**4.47**	**488**	**810**	**2.57**
Butanol	Band broadening and retardation especially in case of EZT				
THF	3.39	5.15	464	756	2.05
Acetonitrile	Incomplete separation				
Type of internal phase					
Octanol	**2.64**	**4.47**	**488**	**810**	**2.57**
Di-isopropyl ether	2.81	4.68	339	749	2.25
Ethyl acetoacetate	2.90	7.77	310	596	4.92

**Table 2:** Performance data for the determination of the studied drugs by the proposed MELC method.

Parameter	SIM	EZT
Concentration range (*μ*g/mL)	0.5–40	1–50
Correlation coefficient	0.9999	0.9999
Slope	68.35	41.93
Intercept	−3.07	−10.55
LOD (*μ*g/mL)	0.15	0.17
LOQ (*μ*g/mL)	0.47	0.53
*S*_*y*/*x*_	8.35	4.81
*S*_*a*_	3.18	2.21
*S*_*b*_	0.22	0.11
% RSD	0.68	0.58
% Er	0.26	0.22

**Table 3:** Assay results for the determination of the studied drugs in pure form, by the proposed MELC method and the reference method.

Parameter	Proposed method		Reference method [30]	
	SIM	EZT	SIM
$\overset{-}{X}\pm \text{SD}$	100.35 ± 0.68	99.83 ± 0.57	99.98 ± 0.61	100.05 ± 0.36
*t-*value	0.95 (2.26)	0.70 (2.26)		
*F*-value	1.85 (4.76)	3.75 (4.76)		

Each result is the mean recovery of three separate determinations.

Figures between brackets are the tabulated *t*- and *F*-values at *P* = 0.05.

**Table 4:** Accuracy and precision data for the determination of the studied drugs by the proposed MELC method.

	SIM concentration (*μ*g/mL)			EZT concentration (*μ*g/mL)		
	10.0	20.0	30.0	20.0	30.0	40.0
Intraday						
$\overset{-}{X}$	100.47	100.01	99.70	100.20	99.84	99.63
±SD	0.51	0.27	0.62	0.60	0.44	0.57
% RSD	0.51	0.27	0.62	0.60	0.44	0.57
% Error	0.30	0.16	0.36	0.35	0.25	0.33
Interday						
$\overset{-}{X}$	100.12	99.97	99.89	100.04	100.08	99.81
±SD	0.37	0.30	0.84	0.73	0.58	0.46
% RSD	0.37	0.30	0.84	0.73	0.58	0.46
% Error	0.21	0.17	0.49	0.42	0.33	0.26

Each result is the mean recovery of three separate determinations.

**Table 5:** Assay results for the determination of the studied drugs in their synthetic mixture using the proposed MELC method.

Parameter	Proposed method		Reference method [30]	
	SIM	EZT	SIM
$\overset{-}{X}\pm \text{SD}$	99.94 ± 0.55	99.71 ± 0.60	100.18 ± 0.36	99.92 ± 0.35
*t-*value	0.65 (2.57)	0.55 (2.57)		
*F*-value	2.32 (9.55)	2.87 (9.55)		

Each result is the mean recovery of three separate determinations.

Figures between brackets are the tabulated *t*- and *F*-values at *P* = 0.05.

**Table 6:** Assay results for the determination of the studied drugs in their coformulated tablets using the proposed MELC method.

	Parameter	Proposed MELC method		Reference method [30]	
		SIM	EZT	SIM
Alkor 20 plus tablets	$\overset{-}{X}\pm \text{SD}$	99.73 ± 0.19	99.69 ± 0.74	99.94 ± 0.43	100.04 ± 0.50
	*t-*value	0.76 (2.78)	0.69 (2.78)		
	*F*-value	5.35 (19.00)	2.18 (19.00)		

Alkor 40 plus tablets	$\overset{-}{X}\pm \text{SD}$	99.85 ± 0.26	99.92 ± 0.79	99.74 ± 0.42	99.66 ± 0.48
	*t-*value	0.41 (2.78)	0.49 (2.78)		
	*F*-value	2.69 (19.00)	2.73 (19.00)		

Each result is the mean recovery of three separate determinations.

Figures between brackets are the tabulated *t*- and *F*-values at *P* = 0.05.
